# How Do Anticipated and Self Regulations and Information Sourcing Openness Drive Firms to Implement Eco-Innovation? Evidence from Korean Manufacturing Firms

**DOI:** 10.3390/ijerph16152678

**Published:** 2019-07-26

**Authors:** Cheon Yu, Junghoon Park, Yun Seop Hwang

**Affiliations:** 1 Department of International Commerce, Finance, and Investment, Kyung Hee University, Seoul 02447, Korea; 2 Narendra Paul Loomba Department of Management, Zicklin School of Business, Baruch College, City University of New York, New York, NY 10010, USA; 3 Department of International Business and Trade, Kyung Hee University, Seoul 02447, Korea

**Keywords:** eco-innovation, institutional theory, anticipated regulation, self-regulation, information sourcing openness, multivariate probit model, zero inflated negative binomial model

## Abstract

Building upon institutional theory and the concept of openness to external sources in terms of breadth and depth, this study investigates the following three understudied drivers of eco-innovation in terms of external and internal factors: Anticipated regulation and self-regulation as external drivers, and information sourcing openness comprised of breadth and importance as internal drivers. Toward this end, this study employs a sample of 1824 Korean manufacturing firms collected from the Korean Innovation Survey 2010, which is compatible with the Oslo Manual and the Eurostat Community Innovation Survey (CIS). The current research adopts a multivariate probit model for the nine binary outcome variables and a zero-inflated negative binomial (ZINB) regression model for a count variable. It is found that, both anticipated regulation and self-regulation positively affect eco-process innovation and eco-product innovation across all of the nine eco-innovation types. The empirical findings on the effects of the breadth of external sources and the importance of used information acquired from external sources for innovative activities indicate that both the breadth and the importance have positive impacts on the number of types of eco-innovation with which a firm is engaged.

## 1. Introduction

Large-scale societal challenges related to the natural environment, such as climate change and loss of biodiversity, have become major concerns for firms, regulators, intergovernmental actors, and civil society, due to the significant consequences of these challenges [1,2,3]. Under these circumstances, securing resources and capabilities to address such challenges has been crucial for businesses to bolster their competitiveness [4], which is normally accompanied by increased demands and pressures from different types of stakeholders on a firm’s pursuit of eco-innovation [5]. While there have been many studies identifying the drivers of eco-innovation of firms in terms of external and internal organizational factors [5,6,7,8,9,10,11,12], some of the determinants examined by the previous studies have yet to be delineated with a theoretical explanation and empirical evidence for the causality between such drivers and eco-innovation activities of firms. To fill this gap, this study investigates the following three understudied drivers of eco-innovation, in order to see how the three factors drive firms to be engaged with eco-innovation: Anticipated regulation and self-regulation as external factors, and information sourcing openness as an internal organizational factor.

To this end, this study draws on institutional theory [13,14,15] and the concept of firms’ external search openness suggested by Laursen and Salter [16] to shed light on the associations between firms’ engagement with eco-innovation and the three underexamined drivers. First, institutional theory suggests that firms conform to pressures from institutions around them in order to obtain social legitimacy [14,17]. Thus, institutional theory provides reasoning on how firms act when they face institutional pressures from stakeholders such as regulators and competitors [18]. Second, Laursen and Salter [16] categorized external information or knowledge collected for innovation activities in terms of breadth and depth and analyzed how the openness of external search sources affects firms’ innovation performance. In their study, external search breadth is defined as “the number of external sources or search channels that firms rely upon in their innovative activities” (p. 134), and external search depth refers to “the extent to which firms draw intensively from different search channels or sources of innovative ideas” (p. 136). Therefore, it is expected that institutional theory and the concept of external search openness may provide a theoretical lens for the impacts on firms’ engagement with eco-innovation of anticipated regulation and self-regulation, on one hand, and of information sourcing openness, on the other hand.

Furthermore, previous studies on the determinants of eco-innovation have been limited in terms of geographical concern. From a geographical point of view, much of the research is focused largely on developed countries such as Germany [6,7,19], Spain [20,21,22], UK [23,24], Ireland [9], and European countries [25,26]. Asian countries and regions, such as China [8,10,12] and Taiwan [27,28], have recently been studied, but there has been little research conducted on Korea, where pollution prevention and climate change issues have become the center of public interest. To keep pace with such public requests, the Korean government has proactively implemented policies relevant to environmental issues, mainly focusing on climate change. In 2009, according to the five-year National Plan for Green Development, the Korean government set the goal of “30% reduction of greenhouse gas (GHG) compared to business as usual (BAU) by 2020.” The government has tried to achieve the goal by introducing “the Primary Law for Low-Carbon Green Growth” in 2010 and “the Goal Management System of Green-house Gas and Energy” in 2011 and implementing “Carbon Emission Trading System” in 2015. These circumstances have led Korean firms to adapt to new environmental regulations and systems. Thus, a business context in Korea not only provides further insights to the literature on drivers of eco-innovation, but also strengthens external validity to confirm the drivers that have been identified in the previous studies.

This study contributes to the existing literature on what determines firms to be engaged with eco-innovation in three ways. First, most prior studies delve into figuring out the effects of existing regulatory pull or push factors on firms’ implementation of eco-innovation, including existing environmental regulation or taxes, and government subsidiaries. Although there is some prior literature that has examined the effects of anticipated regulation on firms’ pursuit of eco-innovation, the relevant empirical findings are mixed [7,9,25]. In addition, research on the impacts of self-regulation or voluntary agreement on eco-innovation is limited as well [9]. Given these research gaps and the complementary effects between government regulation and industry self-regulation [29], this study takes two understudied institutional factors, namely anticipated regulation and self-regulation, into account and examines how these two factors are associated with business engagement with eco-innovation.

Second, to the best of our knowledge, no studies on eco-innovation have examined information sourcing openness in terms of breadth and importance with a sufficient theoretical explanation and empirical test, despite the important role of external information sources in implementing eco-innovation [7,26,30]. Few prior studies have analyzed how the type of information source impacts eco-innovation [7,26]; however, just examining the effects on eco-innovation of type of information source may not provide further implications on theoretical mechanisms behind the relation between information sourcing openness and firms’ pursuit of eco-innovation. Thus, we expect to contribute to the prior studies on the drivers of eco-innovation by investigating the effects on eco-innovation of information sourcing openness in terms of breadth and importance. The concept of importance here is newly suggested to refer to the level of importance of the information used for innovative activities from external sources, because firms cannot help but consider the level of importance of such information to them. This study expects that firms that are open to external sources in terms of breadth, in addition to absorbing a great deal of important information, will have more success in implementing different types of eco-innovation than those firms that are unconditionally open to external sources, without consideration of the importance level of information.

Third, this study reinforces external validity on the empirical findings on the determinants of eco-innovation demonstrated in the previous studies by employing a sample of Korean manufacturing firms extracted from the Korean Innovation Survey 2010 (KIS 2010). The external validity issue is well-addressed in this study because this work revisits the drivers of eco-innovation that have been frequently addressed in the previous literature by incorporating them into both conceptual and empirical models, including regulatory pull and push factors, market pull and pressure factors, organizational factors in terms of size, firm age, innovation capability, and technology push factors. Additionally, the KIS 2010 is compatible with the Oslo Manual developed by the OECD and the Eurostat Community Innovation Survey (CIS) that have been used in many studies on the drivers of eco-innovation. As a robustness check, this study also uses the total number of types of eco-innovation with which a firm is engaged as an outcome variable, so as to confirm the empirical findings on the impacts of the drivers on the likelihood of firms’ implementing eco-innovation.

The remainder of the paper is organized as follows: Section 2 reviews institutional theory, the concept of external search openness, and relevant literature on drivers of eco-innovation, followed by the research hypotheses. In Section 3, methods covering a research model, data sample, definitions and measurements of the variables, descriptive statistics, and the estimation methods are presented. Section 4 illustrates the empirical findings. Section 5 delineates the conclusions and implications, as well as a future research avenue.

## 2. Theorizing and Formulating Hypotheses

### 2.1. Concepts of Eco-Innovation

This study is focused on firm-specific innovation activities related to products and processes. According to [31], eco-process innovation is defined as a newly-introduced element on the production process of eco-friendly products. And eco-product innovation is defined as an introduction of new or ground-breaking eco-friendly products. These definitions pay more attention to realized results or impacts than motivations [7], which implies that “if innovations lead to positive environmental effects, they are defined as eco-innovations” (p. 2) [26]. Given the definition and the survey questionnaires regarding eco-innovation in the KIS 2010, eco-process innovation is defined and measured by whether firms implement any of the following eco-process innovation types that contribute to the natural environment during production processes: Reduction of material use per output unit in the process, reduction of energy use per output unit in the process, reduction of emitted carbon-dioxide, replacement of polluting and hazardous materials with less environmentally harmful ones, reduction of soil, water, noise, and air pollution, and recycling of waste, water and materials. Conversely, eco-product innovation is defined and measured by whether firms implement any of the following eco-product innovation types with environmental contributions that occur if end consumers use goods or services: Reduction of energy consumption from using concerned products, reduction of water and air pollutants, and improvement of recyclability after use. The current study employs a sample of Korean manufacturing firms to analyze how the drivers affect firms’ pursuit of eco-process and eco-product innovations.

### 2.2. Drivers of Eco-Innovation 

The drivers of eco-innovation have been discussed in various ways. Among the different types of drivers, regulatory factors, market pull factors, technology push factors, and some firm-specific factors, have all long been investigated by a number of scholars [5,7,8,9,10,11,25]. In this study, anticipated regulation and self-regulation are considered as the main predictors. When it comes to the internal factors, information sourcing openness consisting of breadth and importance is considered as a main explanatory variable, and other firm-specific factors from the extant studies, such as technology and organizational capabilities, are also adopted.

#### 2.2.1. External Drivers of Eco-Innovation

**Anticipated Regulation**

Institutional theory provides theoretical reasoning on the role of institutional pressures in firms’ decisions on whether or not to implement eco-innovation. Institutional theorists suggest that institutions consist of “cultural-cognitive, normative and regulative elements that, together with associated activities and resources, provide stability and meaning to social life” (p. 33) [17]. One of the main assumptions of institutional theory is that firms make strategic decisions by paying more attention to normative rationality than economic rationality [32]. Firms internally accept and follow what it is considered to be socially valuable by various stakeholders in institutional systems, which is followed by firms’ managerial activities to acquire social legitimacy [33,34,35]. In line with this logic, firms are likely to forestall anticipated regulation by implementing eco-innovation as a response to shifting regulatory institutions to seek legitimacy.

Traditionally, scholars have insisted that regulation has a negative effect on corporate innovation activities and innovation-related performance [36]. Contrary to this view, Porter and Van der Linde [4] argue that firms actively conduct innovations to prevent environmental pollution under a stricter future regulation. Pre-emptive responses for anticipated regulation allow firms to save costs that are possibly incurred in the future, by applying their technologies related to the anticipated future regulation in advance to being engaged with producing goods or services [37]. For example, firms that succeed in innovation beforehand may prevent their current or potential competitors from easily entering the market by utilizing their own competitive advantage, by establishing an environmental practice or standard within an industry [38]. Although there still exist organizational coordination problems [39], firms may collect information about the anticipated regulation and take strategic actions if they are aware of these advantages.

Recent empirical findings have demonstrated that anticipated regulation spurs eco-innovation [7,9,40]. The path on how anticipated regulation affects eco-innovation is explained in two ways. First, there is a firm’s recognition path. If a firm anticipates that a new regulation will be imposed in the future and recognizes it either as a threat or an opportunity, then the firm will take strategic actions in response to the expected regulation. That firm may reset its strategic direction and frame according to the new institutional conditions [41]. The second path is about late movers’ learning from the actions of first movers [42]. First movers’ proactive responses to current regulations may lead late movers to pursue environmental practices such as eco-innovation activities. Thus, late movers may be able to respond more actively to anticipated regulation than the first movers, later on. Through these two paths, anticipated regulation is expected to accelerate firms’ implementation of eco-innovation. Based on the discussion above, we propose the following hypothesis.

**Hypothesis** **1.** *Anticipated regulation will be positively associated with the likelihood of firms’ implementation of eco-innovation.*

**Self-Regulation**

Another external factor that may influence firms’ pursuit of eco-innovation is self-regulation within an industry. A regulation is generally imposed by the government. On the contrary, stakeholders who are subject to such government regulations may voluntarily regulate their own activities. Self-regulation includes establishing financial exchanges, licensing professionals, setting safety standards, controlling entertainment content, advertising restrictions, and voluntarily reducing pollution [43], which may be continuously changed along with the development of technology and society. Some of the reasoning for how self-regulation impacts firms’ eco-innovation is drawn from institutional theory. Institutional theory is based on bounded rationality [13,14]. In response to uncertain and ambiguous environments, firms are expected to consider mimetic isomorphism as a sort of problemistic search [14,44]. In this regard, if there is a self-regulatory institution of firms in the same industry with the purpose of diffusing best environmental practices such as eco-innovation and sustainable supply chain management, then there is a possibility that a firm within that industry may decide to mimic other peers’ best environmental practices to address natural environment issues because such issues may provide firms with uncertainty. Also, a self-regulatory association of firms may play a role as a coercive force to motivate nonmembers in the industry to act in an environmentally sustainable manner [29], which is in line with a primary logic of institutional theory, namely that firms may act the same to conform to institutional pressures to obtain social legitimacy.

Furthermore, self-regulation is formed under the following conditions: (1) When there is a market failure; (2) When it is hard to adjust the market failure, or the failure is accompanied by enormous costs; (3) When self-regulation is more efficient than government regulation [45,46]. Ogus [46] argues that self-regulation is advantageous for the following reasons: First, self-regulation agencies have many experts and experiences of technological innovation trials in a certain area. They may also establish standards with less information costs, so firms under self-regulation have greater innovation potential; Second, firms can reduce costs in monitoring and enforcement through creating reliability among stakeholders. Third, self-regulation allows firms to reduce costs for enforcement and standard revision because self-regulation is somewhat less formal than government regulation. Thus, self-regulation has a positive impact on firms’ pursuit of eco-innovation activities because voluntary regulation enables firms to reduce burden from forceful government regulation, to boost competitiveness on environmental sustainability practices, and to appeal to the market effectively with their environmentally responsible behaviors [47]. It is predicted that firms benefit from self-regulation on account of the regulatory flexibility, the pre-emption of existing and expected regulations. Given the discussion above, the following hypothesis is formulated:

**Hypothesis** **2.** *Self-regulation will be positively associated with the likelihood of firms’ implementation of eco-innovation.*

#### 2.2.2. Internal Drivers of Eco-Innovation

**Information Sourcing Openness**

Prior studies on firms’ innovation strategies suggest that innovation performance is improved if firms are open to external sources to draw information [16,48]. When firms seek information for innovative activities, they not only consider the types of external sources, but also take the importance of information from such sources into consideration. Firms that use a broad range of external sources of information may have more opportunities to implement different types of innovation than those that exploit less sources. In this regard, Laursen and Salter [16] suggested the concept of openness to external sources of information on innovative activities in terms of breadth and depth. For the breadth of external sources, Ghisetti et al. [49] suggested two reasons why firms need a broad external information sources for eco-innovation. First, it is difficult for firms to respond to various environmental changes such as climate change with only their own internal resources and capabilities. Second, eco-innovation usually requires a huge volume of information to achieve multiple objects simultaneously from the improvement of the productivity and quality of innovation to the achievement of environmental targets. Rennings and Rammer [19] demonstrated that firms use relatively diverse sources of information to obtain eco-innovation outcomes. Horbach et al. [30] also argued that eco-innovation needs more external information sources. Additionally, De Marchi and Grandinetti [50] argued that the information obtained from external partners such as research institutions, colleges, or competitors is more important to eco-innovation than to other types of innovation.

Furthermore, firms also need professional and in-depth information in order to carry out innovative activities. In this study, given the concept of depth provided by Laursen and Salter [16], we use the concept of importance of used information obtained from external sources, instead of depth. To some extent, importance is similar to depth, in that both concepts are geared towards describing firms’ purpose of improving their competitiveness by utilizing professional information from external sources. However, the importance assesses the level of significance of used information for innovative activities acquired from external sources, while the depth refers to the degree of specialty of, and collaboration with, partner firms. Rapid and complex changes in the external environment may urge firms to gather important information or knowledge to firms from external sources [48]. A firm is more likely to implement eco-innovation if the firm acquires a great amount of important information for eco-innovative activities from various external sources. According to the discussion above, the following hypotheses are proposed.

**Hypothesis** **3.** *The broader external information sources firms utilize, the more likely the firms implement eco-innovation*.

**Hypothesis** **4.** *The more important information obtained from external sources firms recognize, the more likely the firms implement eco-innovation.*

## 3. Methods

### 3.1. Research Model

**Figure 1:**
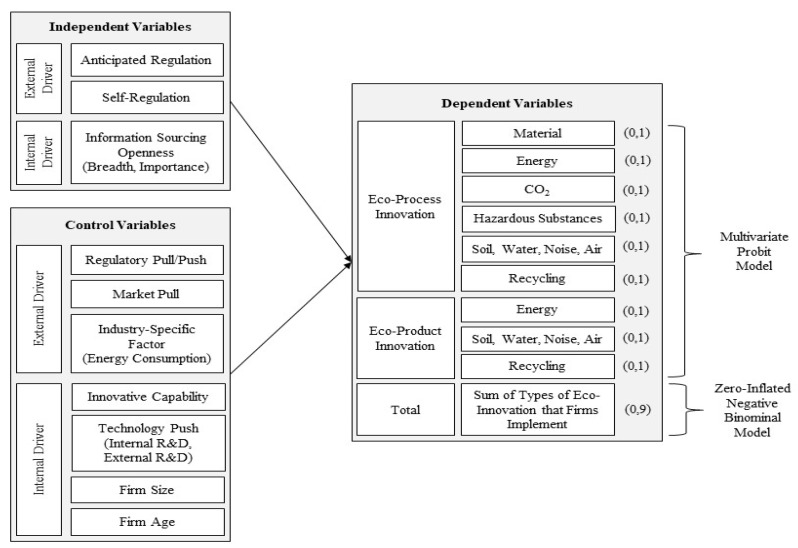
Research Model.

A research model of this study is presented in Figure 1. The dependent variables are categorized into eco-process innovation, eco-product innovation, and the sum of the types of eco-innovation that firms implement as a measurement of the extent to which firms are engaged in the implementation of eco-innovation. Eco-process innovation consists of six types and eco-product innovation is comprised of three types. Whether or not a firm implements a certain type of eco-innovation is measured in binary variables. The three independent variables consist of two external drivers relevant with institutional contexts (anticipated regulation and self-regulation) and one internal driver related to openness to external sources of information for innovative activities in terms of breadth and importance.

Regarding control variables, this study considers both external and internal factors that have been examined in prior studies. External factors are market pull, regulatory pull and push, and an industry-specific factor in terms of energy consumption of an industry. According to the demand-pull factor hypothesis, market demands promote technological innovations [51,52]. Firms are likely to gain competitive advantage by pursuing eco-innovation if they are aware of any market demands on eco-friendly goods or services triggered by the need for energy saving and environmental preservation [40,53,54,55]. Regulatory push and pull factors are also considered as main drivers of eco-innovation [5]. There is a conventional view that mandatory regulation imposed by government (regulatory push) hampers economic growth, due to a rise in cost burdens [36,56,57,58]. In this case, firms may try to avoid an increase in costs, followed by reducing innovative activities which normally require firms to spend much capital. However, there is a contradictory argument that the government’s environmental regulation is regarded as a crucial driver that leads firms to implement eco-innovation [7,59]. A stricter regulation drives firms to consider pursuing eco-innovation to reduce environmental pollutants, which ultimately enables firms to boost their profits above costs [4]. In addition, governmental supporting policies (regulation pull factor) have been discussed as a driver of eco-innovation with a positive impact on firms’ adoption of eco-innovation in prior studies [6,60].

Conversely, internal factors considered as control variables in the current study are innovative capability, technology push factor, firm size, and firm age. Eco-innovation requires firms to accumulate more professional and in-depth knowledge compared to other types of innovation [61,62], which implies that firms’ innovative capabilities on the other innovations may suffice to implement eco-innovation. General innovation capability includes technological innovation capability for products and processes and non-technological capability for markets and organizations. Normally, a high level of general innovation capability lead firms to consider implementing eco-innovation. A technology push factor such as research and development (R&D) investment is also regarded as an important driver of eco-innovation. Firms’ investments in R&D may produce new technological knowledge and promote technological innovation by strengthening their internal capabilities that enable them to assess, assimilate, and exploit relevant knowledge or information obtained from external sources [63]. In this regard, Horbach [6] presented an empirical finding, that improved technological capabilities by R&D activities are positively associated firms’ implementing eco-innovations.

### 3.2. Data Sample

The data employed for an empirical analysis in the current study are the “Korean Innovation Survey 2010 (KIS 2010): Manufacturing sector.” The KIS 2010 was conducted by the Science and Technology Policy Institute (STEPI) to gather information on Korean manufacturing firms’ innovation activities covering periods from 2007 to 2009 in accordance with the Oslo Manual developed by OECD and Eurostat Community Innovation Survey (CIS). The KIS 2010 is approved by Statistics Korea (KOSTAT) and recognized to have high reliability, validity, and international comparability. The population of the KIS 2010 consists of 41,485 Korean manufacturing firms with more than 10 employees, that was established before 2007, and the final sample of the KIS 2010 is comprised of 3,925 firms selected through a stratified sampling method. The KIS 2010 received a response rate of 51.03% (3,925/8,792). The 9th Korean Standard Industrial Classification (KSIC), revised in 2007, was adopted to classify the industry of the KIS 2010 sample firms. Our sample consisting of 1824 firms chosen for an empirical test after the deletion of missing data.

Of the 23 variables, only the variable for the energy consumption of an industry was calculated using the data from a 2013 government report on GHG emissions by industry covering the statistics on GHG emissions and energy consumption in 2012, provided by the National GHG Emission Total Information System (NETIS) and the Korea Energy Agency (KEA). Because the KIS 2010 contained the KSIC code at the group level (denoted by 3 digits), energy consumption data at the same industry categorization level has only become available since the 2012 data. Although the period covered by the KIS 2010 data does not match with the one of energy consumption data, this study employs the industry energy consumption data in 2012, because the energy consumption pattern by industry appears to remain stable from 2007 to 2012. Furthermore, using the energy consumption data by industry at the division level (denoted by 2 digits) may not reflect the differences in these statistics across the manufacturing sectors at the group level (3 digits). The definitions and measurements of the 23 variables are summarized in Table 1.

### 3.3. Descriptive Statstics

Table 2 reports descriptive statistics covering the observations, means, maximum, minimum, and standard deviations for all the variables used in the study. The data consist of a total of 1824 observations for all the variables. The means of the dependent variables (D1 to D9) present the proportion of firms in the sample engaged in each type of eco-innovation. Among the six types of eco-process innovation, D6 (34.9%; Recycle of waste, water, and materials) and D4 (31.8%; Replacement of polluting and hazardous materials with less environmentally harmful ones) are the most common types implemented by Korean manufacturing firms in the sample. Of the three types of eco-product innovation, D7 (31.6%; Reduction of energy consumption) is the most common type pursued by firms in the sample. The mean of D9 indicates that the sample firms implemented 2.6 types of eco-innovation out of the nine types, on average, between 2007 and 2009. The means of the four predictor variables are as follows: Anticipated regulation 20.2 (%), Self-Regulation 16.5 (%), Breadth 6.852 (types), Importance 2.944 (degree). It is noted that the KIS 2010 included a questionnaire on the type of information sources for firms’ innovations as follows: Internal sources, group affiliates, suppliers, customers, competitors/other firms, trade associations/unions, newly hired employees, private service firms (consulting firms, private research institutions), universities, public research institutions, conferences/exhibitions, peer-reviewed academic journals/books. Because the current study is focused on external information sources, this study considers a total of 11 sources except for internal sources.

Given the KSIC at the division level (23 manufacturing sectors except for manufacture of tobacco products 12; 10–33), 16 sectors, ranging from manufacture of pulp, paper and paper products to manufacture of other machinery and equipment, account for 89.26% of the sample. The other seven sectors (manufacture of coke, briquettes and refined petroleum products: 0.88%, manufacture of beverages: 1.26%, manufacture of wood and of products of wood and cork; except furniture: 1.31%, manufacture of leather, luggage and footwear: 1.59%, printing and reproduction of recorded media: 1.69%, manufacture of wearing apparel, clothing accessories and fur articles: 1.98%, and manufacture of other transport equipment: 2.03%) account for a relatively small fraction of the sample.

Except for the correlations among the 10 dependent variables, the correlations among the study variables are generally low to moderate, suggesting that there is a low possibility of facing multicollinearity issues with this set of variables. This is confirmed by the analysis of variance inflation factor (VIF) of the regression model using all the variables. None of the VIFs are greater than 1.69, suggesting that there is no serious multicollinearity problem in the regression model, since the maximum VIF value does not exceed 10.

### 3.4. Analytical Model

There are two kinds of dependent variables in the research model. One is a binary variable, and the other is a count variable. The former is measured by whether or not a firm implements a certain type of eco-innovation out of the nine types covering the six types of eco-process innovation and the three types of eco-product innovation. The latter is the sum of eco-innovation types with which a firm is engaged. Given the first dependent variables, this study employs a multivariate probit model to analyze the effects of the predictors and the control variables on firms’ implementation of the nine eco-innovation types simultaneously. In this study, the first type of dependent variable will have 0 if a firm does not engage in a certain type of eco-innovation and will be 1 if a firm engages in a certain type of eco-innovation. A multivariate probit model is a generalizable form of probit model to estimate several correlated binary outcomes jointly. In this research, a multivariate probit model is used for a simultaneous estimation of the nine types of eco-innovation. The existing evidence on the likelihood of complementarities among different types of innovation shows that unobserved firms’ characteristics may jointly influence the nine types of eco-innovation [25]. If these correlations were neglected, parameter estimates would be biased and inconsistent. A multivariate probit model is written as follows:

(1) $$y_{im}^{*}=\beta_{imo}+\beta_{im1}x_{1}+\beta_{im2}x_{2}+\cdots +\beta_{imp}x_{p}+\varepsilon_{im}$$

(2) $$y_{im}=1\;if\;y_{im}^{*}>0\;and\;0\;otherwise.$$

where *i* represents an individual firm; $y_{im}$ are the nine types of eco-innovation measured as binary variables (*m* = 1, 2, …, 9); $x_{p}$ are independent variables and control variables (*p* = 1, 2, …, 13); and $\varepsilon_{im}$ are error terms distributed as multivariate normal, each with a mean of zero, and variance–covariance matrix V, where V has value the of 1 on the leading diagonal and correlations $\rho_{jk}=\rho_{kj}$ as off-diagonal elements.

Furthermore, the current study estimates a zero-inflated negative binomial (ZINB) regression model for the sum of types of eco-innovation that a firm implements. There are two basic methods for modeling count variables with an excess of zero counts: A zero-inflated Poisson (ZIP) model and a ZINB model. To use a Poisson model, the following two requirements should be satisfied: A Poisson distribution and a constraint of the variance being equal to the sample mean [64]. Conversely, a ZINB model does not have the constraints of a ZIP model. This study ultimately adopts a ZINB model to address excessive zero counts in the dependent variable, because a zero inflated model is suitable for situations in which data are sampled from a population that has two distinct sub-populations. According to Table 3, a ZINB model is preferred over a ZIP model. Therefore, a ZINB model is used to estimate the determinants of the total number of eco-innovation types that a firm implements.

A ZINB regression model assumes that there are two distinct processes in generating zero outcomes. The results of a Bernoulli trial are used to determine which of the two processes reach a zero response. For an observation i, with probability $\pi_{i}$, the only possible response of the first process is a zero count, and with the probability of (1$-\pi_{i}$), the response of the second process is estimated by a negative binomial model with the mean $\mu_{i}$. The zero counts are generated from both the first and second processes, where a probability is estimated for whether zero counts are from the first or the second process. The overall probability of zero counts is the combined probability of zeros from the two processes. A ZINB model for a response $Y_{i}$ is written as follows:

(3) $$\{\begin{array}{c} P\left(Y_{i}=0\right)=\pi_{i}+\left(1-\pi_{i}\right)\cdot {\left(\frac{k}{\mu_{i}+k}\right)}^{k}\\ P\left(Y_{i}=n\right)=\left(1-\pi_{i}\right)\cdot \frac{\Gamma \left(Y_{i}+k\right)}{\Gamma \left(k\right)\Gamma \left(Y_{i}+1\right)}\cdot {\left(\frac{k}{\mu_{i}+k}\right)}^{k}\cdot {\left(1-\frac{k}{\mu_{i}+k}\right)}^{Y_{i}} \end{array}$$

where *k* is the over dispersion parameter; Γ is the gamma distribution, and *n* is a natural number larger than 0. We can model $\pi_{i}$ and $\mu_{i}$ as a function of a set of explanatory variables. For $\pi_{i}$, it is common to use a logistic regression with a logit link function, as it describes a binomial process:

(4) $$logit\left(\pi_{i}\right)=e^{\alpha +\beta_{1}X_{1}+\beta_{2}X_{2}+\cdots +\beta_{n}X_{n}}$$

where α is the intercept, $\beta_{1}$ … $\beta_{n}$ are the model parameters to estimate, and $X_{1}$ … $X_{n}$ are a set of independent variables. We can also model the dependence of $\mu_{i}$ on a different (or same) set of explanatory variables with the aid of a log link function:

(5) $$log\left(\mu_{i}\right)=\lambda +\delta_{1}Z_{1}+\delta_{2}Z_{2}+\cdots +\delta_{n}Z_{n}$$

where λ is the intercept, $\delta_{1}$ … $\delta_{n}$ are the model parameters to estimate, and $z_{1}$ … $z_{n}$ are a set of independent variables.

## 4. Empirical Findings and Discussion

Table 4 demonstrates the empirical results of a multivariate probit model that is employed to analyze a sample of 1824 Korean manufacturing firms to examine the effects of drivers on the nine types of eco-innovation simultaneously. The corresponding Wald tests analyzing the explanatory power of the entire model indicate that the null hypothesis of all parameters of the explanatory variables being zero is clearly rejected at all common levels of significance for the nine independent probit models. All 36 of the rho terms with positive values at the 1% significance level indicate that a multivariate probit model better predicts the nine response variables than nine separate probit models do, because the results support the rejection of the assumption that the error terms across the nine individual models are not correlated. These results confirm the evidence that unobserved firms’ characteristics have overall effects on the nine types of eco-innovation [25].

The last column in Table 4 reports the findings estimated by a ZINB model, consisting of a logit model predicting certain zero responses and a negative binomial model predicting count responses as a robustness check. The signs of coefficients for most of the variables remain consistent with the ones produced by the multivariate probit model. First, the inflated model uses the five variables that are expected to predict the likelihood of being in the certain zero group (i.e., eco-innovation = 0). In this model, this study considers the following well-established drivers of eco-innovation in terms of external and internal drivers [5,11]: Existing environmental regulations or taxes (Pre-Regu), government subsidies (Subsidy), market demands for eco-innovation (Mkt-Pull), innovation capability (Inno-Capa), and firm size (Firm Size). The coefficients for Pre-Regu, Subsidy and Mkt-Pull are not found to be statistically significant. Conversely, Inno-Capa and Firm Size have negative coefficient values. The results imply that the greater innovative capabilities that a firm has, the more likely it is that the firm implements eco-innovation, and the larger the firm is, the more likely it is that the firm is engaged with eco-innovation.

Second, a negative binomial model predicts the count responses with a range from 1 to 9. All the 13 variables that are used to estimate the multivariate probit model are considered for the negative binomial model. For the independent variables, both external drivers (anticipated regulation and self-regulation) and internal drivers (breadth and importance) have positive coefficient values at the 1%, 1%, 1%, and 10% significance level, respectively. When it comes to the interpretation of a coefficient, if both a firm that was motivated to implement eco-innovation by anticipated environmental regulations and a firm that was not motivated by such regulations implement at least one type of eco-innovation (i.e., not certain zeros), the firm that is motivated by anticipated regulations is likely to implement more types of eco-innovation than the firm that is not motivated by such regulations while holding the other predictors fixed. For the control variables, of external factors, existing regulations, government subsidies, and market demands for eco-innovation have positive impacts on the outcome variable at the 1% significance level. Energy consumption of an industry is not found to be statistically significant. Of the internal drivers, innovative capabilities and firm size have positive effects on the response variable at the 1% and 10% level, respectively. Internal R&D investments have a negative impact on the dependent variable at the 5% level. External R&D investments and firm age are not found to be statistically significant.

### 4.1. The Empirical Findings on the External Factors

Both anticipated regulation and self-regulation has positive impacts on the likelihood of firms’ implementing all the nine types of eco-innovation at the 1% significance level. Therefore, it is concluded that both Hypotheses 1 and 2 are supported. In particular, the results regarding the effects of anticipated regulation on eco-innovation are somewhat different to the empirical findings suggested by a few prior studies [7,9,25,61]. For instance, Triguero et al. [25] suggested that future regulation does not have impacts on either eco-process or eco-product innovation, while Doran and Ryan [9] provided an empirical result showing that expected regulation only has significant impacts on two types of eco-process innovation (replacement of polluting and hazardous materials with less environmentally harmful ones, and reduction of soil, water, noise, and air pollutants) and one type of eco-product innovation (reduction of soil, water, noise, and air pollutants). These results indicate that firms make efforts to gain social legitimacy by pre-empting any future shifts in environmental regulations imposed by the government. Also, firms may decide to implement environment-related innovation activities pre-emptively in response to predicted regulations to obtain competitive superiority by differentiating their products and cutting down expenses [37,38], which implies that the argument by Porter and Van der Linde [4] can be applied to the Korean business context.

For the impacts of self-regulation, the empirical findings show that firms that are willingly engaged in a voluntary agreement within an industry are more likely to implement all nine types of eco-innovation, than those that are not engaged with such a voluntary agreement. These results are aligned with the findings provided by Doran and Ryan [9]. They suggested that voluntary agreements have positive influences on all the nine types of eco-innovation as well. Given that a self-regulatory institution of firms has a purpose of diffusing best environmental practices [29], firms within that industry may be likely to refer to or mimic the best practices such as an effective eco-innovation conducted by other firms engaging in a voluntary agreement in order to cope with natural environment-related challenges. According to the argument by Nash and Ehrenfeld [65], eco-innovation activities can be realized under the circumstance of non-public control, and that voluntarily engaging firms are more likely to exploit eco-innovation than non-voluntary firms. Given the empirical findings, it is predicted that voluntary conventions may play a role in promoting eco-innovations of Korean firms, despite the vulnerability of voluntary conventions, namely that such agreements may be loosely operated compared to government regulations due to no explicit punishments for a free-ride or opportunistic behavior.

### 4.2. The Empirical Findings on the Internal Factors

The results on the impacts of information sourcing openness in terms of breadth and importance vary across the nine types of eco-innovation. Of the six types of eco-process innovation, both breadth and importance positively affect the reduction of material consumption per output unit (D1) and reduction of energy consumption per output unit (D2). Among the three types of eco-product innovation, only breadth has positive impacts on reduction of soil, water, noise, and air pollution (D8) and improvement in recyclability after product use (D9). Therefore, both Hypothesis 3 and 4 are partially supported. These findings may be in line with a few prior studies [7,26] that indicated the different impacts of types of information sources on firms’ implementation of eco-innovation. These findings imply that firms may decide on how broadly they use external sources to obtain information depending on the types of eco-innovation that they would like to implement or pursue.

Interestingly, when it comes to the effects of breadth and importance on the number of types of eco-innovation with which firms are engaged, both variables have positive impacts on the response variable. The broader a range of external information sources for innovative activities a firm has, the more types of eco-innovation a firm is predicted to implement. This result implies that if firms want to implement different types of eco-innovation simultaneously, they may need to secure different external sources for information, as well as take the importance of obtained information from such sources into account. With regard to the Korean business context, Korean firms make efforts to strengthen innovative competitiveness by absorbing information from various external. In a study on the effects of external information search on the Korean ICT sector, Hwang and Lee [66] found that external information search is relevant to incremental innovation and productivity. From the point of supply chain management, Woo et al. [67] investigated communication capability and external green integration for the financial and green performance of construction providers in Korea. These results show that the greater capability a firm shows in sharing information with other organizations, the more likely it is for that firm to acquire superior positions in taking environmentally cooperative actions, in making green cost reduction, and in securing improved competitiveness.

### 4.3. The Empirical Findings on the Control Variables

The results regarding the effects of regulatory pull/push factors on eco-innovation are the same as what the previous studies demonstrated [7,59]. Both present regulation and governmental subsidiaries positively affect the likelihood of firms’ implementing the nine types of eco-innovation at the 1% significance level. For a market-pull factor, higher demands on eco-friendly products have positive influences on all nine types of eco-innovation. This result is consistent with the empirical findings presented by Kammerer [40]. Regarding an industry-specific factor, energy consumption of an industry has positive impacts on four types of eco-process innovation out of six (D2, D3, D5, and D6), but deem to have no significant effects on any types of eco-product innovation. If an average energy consumption within an industry is higher, the likelihood of the firms’ being engaged with eco-innovation is higher in areas including reduction of energy consumption per output unit (D2), reduction of CO_2_ emissions (D3), reduction of soil, water, noise, and air pollution (D5), and recycle of waste, water, and materials (D6). Firms in a high energy consumption industry are likely to focus more on the benefits from cutting down their energy consumption rather than abating consumers’ energy consumption occurred by product use. Furthermore, in manufacturing processes, such firms would concentrate more on innovative activities that are likely to bring reduction outcomes in a relatively short period of time. Thus, firms are less likely to implement types of eco-process innovation that may require a substantial amount of investments and research activities over a long term, such as the replacement of pollutants or hazardous matters with less harmful ones. These findings are consistent with the ones provided by Horbach [6] and Machiba [68].

For internal factors as control variables, innovation capabilities have significant positive effects on the likelihood of firms’ implementing all the nine types of eco-innovation (D3 and D5 at the 5% significance level; the other seven types at the 1% level). This result is confirmed by the empirical finding produced by the ZINB model. As a technology push factor, internal R&D investments have negative effects on the recycling of waste, water, and materials (D6) and improvement in recyclability after product use (D9). The reasoning behind this result may be attributed to the fact that business R&D investments normally have a negative effect on the productivity growth in the short run [69]. Guellec and de la Potterie [69] suggested that it takes some time for firms to realize productivity growth, firms may have negative impacts from such investments in the short run. In a similar vein, Hwang et al. [70] presented that R&D intensity has a negative relationship with firms’ revenues in the short run. External R&D investments do not have significant impacts on any types of eco-innovation at all. Both firm size and firm age have impacts on certain types of eco-innovation. For firm size, it has positive impacts on reduction of CO_2_ emissions (D3) and reduction of energy consumption (D7), but a negative impact on improvement in recyclability after product use (D9). For firm age, it has a positive impact on reduction of energy consumption per output unit (D2) and reduction of soil, water, noise, and air pollution (D5).

## 5. Conclusions

Drawing on institutional theory and the concept of openness to external sources in terms of breadth and depth, the current study examines the following three understudied drivers of eco-innovation in terms of external and internal factors with the empirical evidence from a large sample of Korean manufacturing firms: Anticipated regulation and self-regulation as external drivers, and information sourcing openness comprised of breadth and importance as internal drivers. First, both anticipated regulation and self-regulation not only positively affect the likelihood of firms’ implementing all the nine types of eco-innovation, but also has positive impacts on the number of types of eco-innovation that firms implement. Second, for information sourcing openness, the breadth of external information sources for innovative activities positively affects the likelihood of firms’ pursuing the four types of eco-innovation. The importance of obtained information from such external sources also has positive impacts on the likelihood of firms’ implementing the two types of eco-innovation. However, both breadth and importance have positive impacts on the number of types of eco-innovation with which firms are engaged. Regarding the methods, in order to efficiently estimate the effects of drivers of eco-innovation, this study employs a multivariate probit model and a zero-inflated negative binomial model to predict the nine types of eco-innovation as binary variables and the number of types of eco-innovation with which a firm is engaged as a count variable, respectively, given the characteristics of the ten outcome variables.

This study presents several policy implications for academic contexts. First, although the prior studies have already confirmed that the government’s environmental regulations promote firms’ pursuit of eco-innovations [4,71], the effects of anticipated environmental regulations have not been addressed much, except for a few studies [9,25]. In this regard, this study investigates how firms respond to anticipated environmental regulations imposed by the government both theoretically and empirically. According to the empirical results, firms are found to consider proactive strategies such as the implementation of eco-innovation rather than reactive actions when they recognize predicted environmental regulations. This implies that if the government sent out a signal for further environmental regulations to firms, firms are likely to recognize such a signal as institutional pressure that they need to address in order to gain social legitimacy, as well as competitive advantage, in the future. In particular, the argument that if late movers take anticipated regulation into consideration as an opportunity to obtain competitive advantages, such firms may try to become leading firms in the market for the future by engaging in active strategies for technological innovations, may strengthen the validity of the empirical findings provided by this study. In addition, if firms clearly see the direction of environmental policies, they can decide on their strategies and respond to anticipated regulatory shifts actively. Therefore, keeping consistency and continuation in the government’s environmental policies is important. But even when firms are aware of such regulatory changes, they may not be immune to environmental uncertainty occurred by regulatory shifts. In this case, firms are more likely to take reactive actions when they perceive great uncertainty. On the other hand, they are expected to pursue proactive environmental strategies if they perceive low uncertainty.

Second, self-regulation is found to a positive impact on firms’ engagement in eco-innovation activities. There has been a lack of concrete discussion on the theoretical mechanism behind the relationship between self-regulation and firms’ implementation of eco-innovation. To fill this gap, this study builds upon institutional theory to explain how self-regulation affects the likelihood of firms’ implementation of eco-innovation, followed by the empirical analyses to support the relevant hypothesis. Given the positive impact of self-regulation on firms’ pursuit of eco-innovation, it is concluded that if firms are engaged with voluntary agreements within an industry, with the purpose of diffusing best environmental practices, they would be motivated to implement eco-innovation by other peers with superior eco-innovation performance or best practices in such voluntary agreements. Also, self-regulation may play a role as an effective substitute for inflexible government regulations [46]. From the government’s perspective, self-regulation is regarded as a conduit for the reduction of external effects caused by the establishment of government rules, as well as the decrease in costs on persuading different parties of stakeholders to agree on the government’s direction. Thus, it is important for the government to lay foundations for an effective self-regulation in the long run through joint efforts with firms.

Third, one of the primary contributions of this study is that it provides theoretical mechanisms and empirical findings on how openness to external sources in terms of breadth and importance may encourage firms to implement different types of eco-innovation. Given that eco-innovation intrinsically has complex development processes and multiple purposes, it is crucial for firms to utilize diverse external sources of information for innovative activities [49,50]. Furthermore, multiple dimensions, such as design, user involvement, product and service, or governance should be taken into consideration in the process of implementing eco-innovation [50]. The positive relations between both breadth and importance and the number of types of eco-innovation that a firm implements indicate that if firms want to achieve a competitive superiority by implementing different types of eco-innovation at the same time, they not only need to exploit a variety of external information sources for eco-innovation, but also consider the importance of obtained information from such sources. As this study measures the breadth and the importance based on the data on external sources for general innovations, it would be interesting to examine the same effects of these variables by employing data specific for external information sources for eco-innovation activities. In addition, a recent study by Zhang et al. [12] offers a relevant clue that green absorptive capacity plays a significant role in the relationship between environmental regulation and firms’ adoption of external knowledge. Consequently, it might be of interest to scholars considering the role of firms’ absorptive capacity, which refers to firms’ ability to effectively use outside information [72,73,74] in determining the importance of used external information from different external sources.

Although this study provides several contributions and implications, it is not without limitations. First, the current study did not fully take into consideration the temporal effects of each of the drivers considered in the empirical analyses. As an avenue for future research, a longitudinal data analysis could be conducted to confirm the results provided by previous studies on the drivers of eco-innovation by considering temporal effects. Second, this study was not able to reflect changes in firms’ behaviors on eco-innovation due to the limited data. If data on interpretative categories, whether anticipated regulation is perceived as either opportunities or threats by firms, are available, this would provide further implications on how firms’ eco-innovation strategies and relevant performance will change, depending on their interpretations of anticipated environmental regulation.

## Figures and Tables

**Table 1:** Definitions and Measurements of Variables.

Variables		Label	Definition	Type	Source
IVs	Anticipated Regulation	Exp-Regu	Predicted environmental regulations or taxes	Binary	KIS 2010
	Self-regulation	Self-Regu	Voluntary conventions or agreements within the industry	Binary	
	Information Sourcing Openness	Breadth	The number of used external information sources for innovative activities	Count	
		Importance	The average degree of importance of used information for innovative activities obtained from external sources (=Sum of the extent to which used information obtained from external sources is important/The number of used external sources)	Ratio	
CVs	Regulatory Pull/Push	Pre-Regu	Existing environmental regulations or taxes	Binary	
		Subsidy	Using subsidiaries from the government or financial benefits related to eco-innovation	Binary	
	Market Pull	Mkt-Pull	Market demands for eco-innovation from the current or future consumers	Binary	
	Energy Consumption	Eng-Cons	The average annual energy consumption of an industry in 2012 =Log (Total energy consumption of an industry in 10^9^ kcal/The number of firms of an industry)	Log	NETIS
	Innovation Capability	Inno-Capa	The number of innovations on products, processes, organizations, and marketing	Count	KIS 2010
	Technology Push	In-R&D	Log (0.1+% of internal R&D expense to sales volumes 2007~2009)	Log	
		Ex-R&D	Log (0.1+% of external R&D expense to sales volumes 2007~2009)	Log	
	Firm Size		Log (The number of full-time employees in 2007)	Log	
	Firm Age		The age of the firm (=2010-the year of establishment)	Count	
DVs	Eco-Process Innovation	D1	Reduction of material consumption per output unit	Binary	
		D2	Reduction of energy consumption per output unit	Binary	
		D3	Reduction of CO_2_ emissions	Binary	
		D4	Replacement of polluting and hazardous materials with less environmentally harmful ones	Binary	
		D5	Reduction of soil, water, noise, and air pollution	Binary	
		D6	Recycle of waste, water and materials	Binary	
	Eco-Product Innovation	D7	Reduction of energy consumption	Binary	
		D8	Reduction of soil, water, noise, and air pollution	Binary	
		D9	Improvement in recyclability after product use	Binary	
	Total Number	D10	D1 + D2 + D3 + D4 + D5 + D6 + D7 + D8 + D9	Count	

**Table 2:** Descriptive Statistics.

Variables	Observations	Mean	Std. Deviation	Max	Min
D1	1824	0.282	0.450	0	1
D2	1824	0.298	0.457	0	1
D3	1824	0.247	0.432	0	1
D4	1824	0.318	0.466	0	1
D5	1824	0.280	0.449	0	1
D6	1824	0.349	0.477	0	1
D7	1824	0.316	0.465	0	1
D8	1824	0.240	0.427	0	1
D9	1824	0.270	0.444	0	1
D10	1824	2.600	3.027	0	9
Exp-Regu	1824	0.202	0.402	0	1
Self-Regu	1824	0.165	0.371	0	1
Breadth	1824	6.852	3.297	1	11
Importance	1824	2.944	0.764	1	5
Pre-Regu	1824	0.158	0.365	0	1
Subsidy	1824	0.054	0.227	0	1
Mkt-Pull	1824	0.338	0.473	0	1
Eng-Cons	1824	0.545	1.712	−2.440	7.688
Inno-Capa	1824	2.746	1.122	0	4
In-R&D	1824	−2.050	0.360	−2.303	2.565
Ex-R&D	1824	−2.259	0.126	−2.303	−0.640
Firm Size	1824	4.346	1.406	1.131	10.292
Firm Age	1824	19.730	14.034	4	94

**Table 3:** Tests and Fit Statistics.

ZIP	BIC = 5914.050	AIC = 5803.874	Prefer	Over	Evidence
vs. ZINB	BIC = 5858.568	dif = 55.481	ZINB	ZIP	Very strong
	AIC = 5742.884	dif = 60.990	ZINB	ZIP	
	LRX2 = 62.990	prob = 0.000	ZINB	ZIP	*p* = 0.000

**Table:** (**a**)

Number of obs = 1824 Wald chi2 (117) = 1983.06 Prob > chi2 = 0.000 Log likelihood = −5789.3644			Eco-Process Innovation					
			D1 Material	D2 Energy	D3 CO_2_	D4 Danger	D5 Soil/Water/Noise/Air	D6 Recycle
IVs	External Factors	Exp-Regu	0.6972 ***	0.8866 ***	0.8197 ***	0.9260 ***	0.8009 ***	0.7427 ***
			(0.0813)	(0.0797)	(0.0812)	(0.0808)	(0.0791)	(0.0812)
		Self-Regu	0.7235 ***	0.7728 ***	0.7790 ***	0.6439 ***	0.7787 ***	0.8929 ***
			(0.0865)	(0.0845)	(0.0872)	(0.0876)	(0.0863)	(0.0862)
	Internal Factors	Breadth	0.0257 *	0.0221 *	0.0193	0.0093	0.0213	−0.0044
			(0.0136)	(0.0131)	(0.0136)	(0.0131)	(0.0131)	(0.0128)
		Importance	0.0929 *	0.1122 **	0.0767	0.0534	−0.0082	0.0176
			(0.0519)	(0.0502)	(0.0517)	(0.0498)	(0.0487)	(0.0480)
CVs	External Factors	Pre-Regu	0.6731 ***	0.5427 ***	0.5876 ***	0.9656 ***	1.0153 ***	0.9943 ***
			(0.0902)	(0.0889)	(0.0897)	(0.0905)	(0.0876)	(0.0896)
		Subsidy	0.8025 ***	0.7789 ***	0.7982 ***	0.8422 ***	0.8155 ***	0.8056 ***
			(0.1347)	(0.1309)	(0.1374)	(0.1424)	(0.1342)	(0.1414)
		Mkt-Pull	1.1959 ***	1.0813 ***	1.0398 ***	1.0751 ***	1.0348 ***	1.0669 ***
			(0.0767)	(0.0744)	(0.0775)	(0.0753)	(0.0758)	(0.0736)
		Eng-Cons	0.0228	0.0406*	0.0637 ***	0.0066	0.0565 ***	0.0801 ***
			(0.0217)	(0.0211)	(0.0216)	(0.0214)	(0.0209)	(0.0207)
	Internal Factors	Inno-Capa	0.1777 ***	0.1023 ***	0.0964**	0.1787 ***	0.0804**	0.1313 ***
			(0.040)	(0.0384)	(0.0398)	(0.0390)	(0.0386)	(0.0377)
		In-R&D	−0.0841	0.0917	−0.0383	−0.1206	−0.1576	−0.3301 ***
			(0.1143)	(0.0991)	(0.1131)	(0.1188)	(0.1167)	(0.1212)
		Ex-R&D	0.0880	−0.0703	−0.1743	0.4359	−0.0083	0.0728
			(0.2924)	(0.2778)	(0.3082)	(0.3071)	(0.3073)	(0.2946)
		Firm Size	0.0508	0.0323	0.1161 ***	0.0454	−0.0062	0.0124
			(0.0342)	(0.0328)	(0.0337)	(0.0334)	(0.0327)	(0.0327)
		Firm Age	0.0006	0.0071 **	−0.0035	0.0011	0.0078 ***	0.0021
			(0.0030)	(0.0029)	(0.0030)	(0.0030)	(0.0029)	(0.0029)
		_cons	−2.8505 ***	−2.6194 ***	−3.3425 ***	−1.7839 ***	−2.5646 ***	−2.4832 ***
			(0.6849)	(0.6480)	(0.7128)	(0.6960)	(0.7059)	(0.6778)

Note. Standard errors are presented in parentheses. * *p*-value < 0.10; ** *p*-value < 0.05; *** *p*-value < 0.01.

**Table:** (**b**)

Number of obs = 1824 Wald chi2 (117) = 1983.06 Prob > chi2 = 0.000 Log likelihood = −5789.3644			Eco-Product Innovation			Total Number Negative Binomial Regression Part (eco-innovation > 0)
			D7 Energy	D8 Soil/Water /Noise/Air	D9 Recycle	Number of obs = 1824 Nonzero obs = 1049 Zero obs = 775 LR chi2 (13) = 185.59 Prob > chi2 = 0.000 Inflation model = logit Log likelihood = −2846.924
IVs	External Factors	Exp-Regu	0.4974 ***	0.6616 ***	0.5123 ***	0.1671 ***
			(0.0807)	(0.0798)	(0.0788)	(0.0380)
		Self-Regu	0.8165 ***	0.6866 ***	0.7677 ***	0.1661 ***
			(0.0851)	(0.0833)	(0.0827)	(0.0392)
	Internal Factors	Breadth	0.0203	0.0242 *	0.0268 **	0.0297 ***
			(0.0129)	(0.0133)	(0.0129)	(0.0069)
		Importance	−0.0255	0.0582	0.0319	0.0456 *
			(0.0487)	(0.0490)	(0.0481)	(0.0252)
CVs	External Factors	Pre-Regu	0.5879 ***	0.6614 ***	0.6534 ***	0.1126 ***
			(0.0882)	(0.0882)	(0.0876)	(0.0420)
		Subsidy	0.7525 ***	0.6405 ***	0.4983 ***	0.2081 ***
			(0.1395)	(0.1328)	(0.1353)	(0.0575)
		Mkt-Pull	1.2199 ***	1.1177 ***	1.0173 ***	0.2477 ***
			(0.0731)	(0.0762)	(0.0726)	(0.0403)
		Eng-Cons	−0.0039	0.0286	−0.0046	0.0166
			(0.0212)	(0.0209)	(0.0205)	(0.0101)
	Internal Factors	Inno-Capa	0.1519 ***	0.1482 ***	0.1322 ***	0.0574 ***
			(0.0376)	(0.0390)	(0.0375)	(0.0205)
		In-R&D	−0.1117	−0.1193	−0.4013 ***	−0.1354 **
			(0.1143)	(0.1194)	(0.1261)	(0.0607)
		Ex-R&D	0.1870	0.4169	−0.2644	0.1298
			(0.2963)	(0.3067)	(0.3310)	(0.1530)
		Firm Size	0.0620 *	0.0334	−0.0622 *	0.0281 *
			(0.0326)	(0.0327)	(0.0319)	(0.0154)
		Firm Age	−0.0020	−0.0001	0.0014	0.0011
			(0.0029)	(0.0029)	(0.0029)	(0.0014)
		_cons	−1.9993 ***	−1.9692 ***	−3.3432 ***	0.4843
			(0.6752)	(0.6965)	(0.7474)	(0.3520)
Inflated (eco-innovation = 0)		Pre-Regu				−26.1298
						(19,138.8200)
		Subsidy				−25.6075
						(31,736.5000)
		Mkt-Pull				−26.3002
						(13,302.1500)
		Inno-Capa				−0.3999 ***
						(0.0768)
		Firm Size				−0.1716 ***
						(0.0642)
		_cons				3.0180 ***
						(0.3387)
/lnalpha						−2.3352 ***
						(0.1604)
alpha						0.0968

Note 1. Standard errors are presented in parentheses. * *p*-value < 0.10; ** *p*-value < 0.05; *** *p*-value < 0.01. Note 2. 36 rho terms (all significant at the 1% level): rho21 = 0.7125, rho31 = 0.4886, rho41 = 0.4665, rho51 = 0.4289, rho61 = 0.3558, rho71 = 0.4496, rho81 = 0.3464, rho91 = 0.4149; rho32 = 0.6673, rho42 = 0.3551, rho52 = 0.4779, rho62 = 0.3690, rho72 = 0.4849, rho82 = 0.3921, rho92 = 0.3707; rho43 = 0.4358, rho53 = 0.5415, rho63 = 0.3139, rho73 = 0.4853, rho83 = 0.4658, rho93 = 0.3108; rho54 = 0.5778, rho64 = 0.3264, rho74 = 0.2929, rho84 = 0.4481, rho94 = 0.3617; rho65 = 0.5232, rho75 = 0.3835, rho85 = 0.7349, rho95 = 0.4340; rho76 = 0.3010, rho86 = 0.4880, rho96 = 0.5004; rho87 = 0.5419, rho97 = 0.5349; rho98 = 0.5350.
